# Contemporary Theory and Research

**Published:** 1893-04-08

**Authors:** 


### Contemporary Theory and Research.

### CREOSOTAL FOR TUBERCULOSIS.

M. Brissonnet, Professor of Medicine at Tours, has produced a new compound strongly advocated as a remedy for tuberculosis, which he calls creosotal. Creosote possesses a strong odour, and a burning taste irritating to the mucous membrane, and is in consequence only able to be administered in weak doses. But by fixing carbonic acid on creosote (obtained from beech) a neutral body is obtained of a sweet and oily flavour, without odour, not irritating to the mucous membrane, and capable of being absorbed in strong doses without fatiguing the stomach. Creosotal is a viscid liquid at an ordinary temperature, but becomes quite fluid under the influence of even moderate heat. Creosote is considered as the most active medicament against tuberculosis, but on account of its caustic properties only slight doses can be imbibed. But since in creosotal the creosote is disguised in a neutral combination, it is confidently hoped that this preparation will result in a considerable advance in the treatment of tuberculosis.

### DISINFECTION AGAINST CHOLERA.

The *Practitioner* has an important article by Dr. Sternberg on "Disinfection at Quarantine Stations, especially against Cholera." An elaborate series of experiments has been made by the Imperial Board of Health of Germany to determine the length of time the cholera spirillum will survive upon various kinds of fruit and other articles of food. "According to Uffelmann the spirillum may survive upon the printed pages of a book for seventeen hours, and upon writing-paper enclosed in an envelope for twenty-three hours and a-half, upon silver and copper coins it only survives for half-an-hour, upon the dry hand for an hour, but not for two hours." Dr. Sternberg concludes with some practical suggestions as to disinfection by heat. "The thermal death-point of the cholera spirillum in a moist condition (bouillon culture) as determined by the writer is 52° C. (125·6° F.), the time of exposure being ten minutes. It is with certainty destroyed in a very brief time by a temperature of 60° C. (140° F.) The demand, therefore, that it shall be subjected for half-an-hour or more to a temperature of 100° C., or to steam under pressure at a higher temperature would certainly be extravagant if the only question related to the destruction of the spirillum by the disinfecting agent. It has been shown by carefully conducted experiments that neither steam nor hot air readily penetrate bundles of piled up heaps of clothing, blankets, &c. The free exposure of such articles in the disinfection chamber is therefore a matter of prime importance. As the cholera spirillum is quickly destroyed by dessication, the question at once arises, "Why not use dry heat instead of steam in cholera disinfection? It is evident that having an exact knowledge of the biological characters of this spirillum we need no longer be controlled in making recommendations relating to its destruction by data relating to anthrex spores or pathogenic bacteria which resist dessication." It is reassuring to learn that free exposure to fresh air and sunshine is one of the most reliable methods of disinfecting articles which have attached to them the cholera spirillum.

### THE USE OF THE SANATORIUM.

M. Bardet considers that the use of hygienic methods tends to take an important place in the healing art, and it is to be supposed that the residence of chronic invalids in sanatoriums will become more and more frequent. The choice of a temperate climate for this purpose permitting residence all the year round is most important. Comparing the weather reports of various favourite resorts M. Bardet declares in favour of Brest for uniformity of climate and high winter temperature, there being fewer frosty days in Brest during the year than at any other spot in France.

### THE OFFSPRING OF MULATTOES.

Dr. Dixon, of Boston, U.S.A., concludes after thirty years of observation that the offspring of mulattoes are the subject of constitutional disease to a greater degree than those of unmixed blood. When confined entirely to their own class, that is, without the admixture of negro or white blood, they scarcely ever reach the fourth generation in descent. Tuberculosis exists to an excessive degree among the descendants of mulattoes; they are inferior in vitality, intelligence, and morality, and show a high rate of mortality. There seems to be no doubt, the author thinks, that the weakness of mulattoes will prevent any large amount of mixture of the negro in the future American citizen.

### RAPID DISINFECTION.

In a paper read at a meeting of the Medical Officers of Schools Association, Mr. J. Brendon Curgenven explained the properties and uses of a disinfectant which certainly appears to be wonderfully efficacious in diminishing the risks of contagion. The preparation in question is known as Tucker's Oleusaban Eucalyptus Disinfectant, and it is claimed on behalf of it that it obviates all necessity for removing fever or small-pox patients to a hospital. It is stated that at Bexley the use of this disinfectant has enabled the medical officer to dispense altogether with an infectious hospital. Out of 157 scarlet fever patients treated with this preparation only two deaths occurred. Many instances are quoted in which by the use of this disinfectant the disease was prevented from spreading, and this even where other children were in direct contact with the patient. Several heads of schools testify to its value in checking epidemics of scarlet fever and diphtheria. Among others, Dr. Alder Smith, the Medical Officer of Christ's Hospital, gives a striking account of the checking of a threatened outbreak of scarlet fever in the school by means of this antiseptic. Mr. Curgenven directs that the patient should be lightly rubbed with the antiseptic night and morning for three days; then each night after a warm bath for seven nights. He should then be free from poison, and unable to infect others. The disinfectant must also be sprinkled over bed and pillow, and diffused in the room by means of a spray producer. Three to six drops of Oleusaban, or of oil of Eucalyptus globulus, may be administered on a lump of sugar three times a day. The only effect which the rubbing produces is a slight smarting of the more sensitive parts of the skin and slight headache. It heightens the colour of the rash and produces a sense of warmth.

